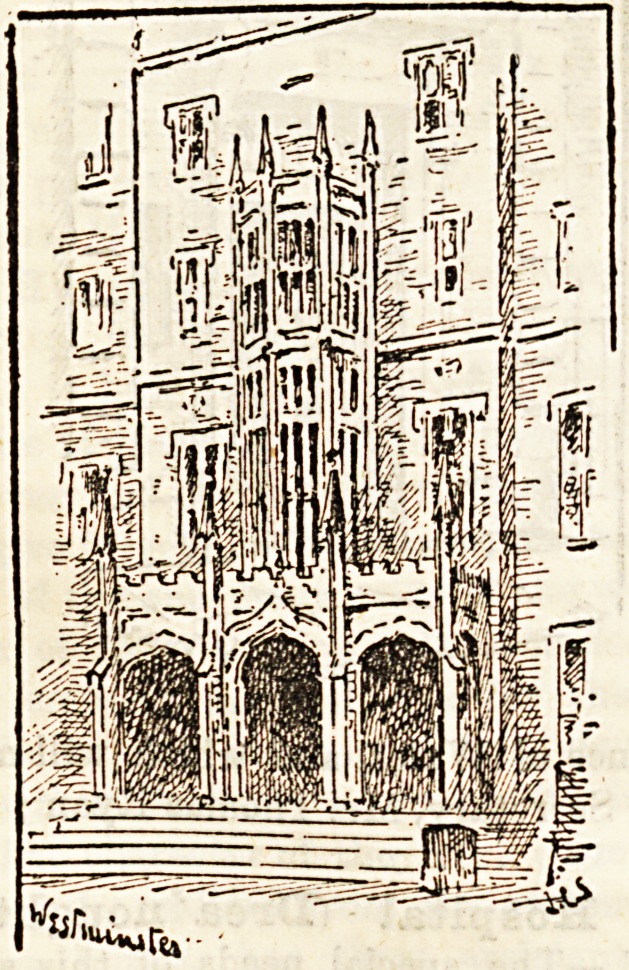# General Hospitals

**Published:** 1892-12-24

**Authors:** 


### SWEET CHARITY'S GUIDE FOR CHRISTMAS GIVERS.

### Being the Christmas Number of "The Hospital."

### GENERAL HOSPITALS.

**Charing Cross Hospital**, Agar Street, West Strand.—This institution is situated in the centre of the most crowded thoroughfares in the metropolis, and has therefore to provide for a greater number of accidents, *i.e.*, urgent cases, than probably any other hospital of its size. All such cases are immediately admitted without delay or difficulty. About 23,500 patients were relieved during the past year, more than two-thirds of which were cases of accident or emergency. Can any stronger appeal be made than this to the many visitors who usually come to London, or to the public generally? We think not. Yet, the need for pecuniary help is great and pressing, and the Council earnestly appeal for donations towards meeting the deficiency between income and expenditure amounting to £6,000, and for annual subscriptions to permanently reduce it. Matron, Miss H. Gordon; Secretary, Mr. A. E. Reade.

**German Hospital**, Dalston, N.E.—The hospital contains 120 beds, which are nearly always occupied, and admits into its wards, without any recommendation, all who are conversant with the German language (without any distinction of nationality or creed), as well as all persons who have sustained injuries from accidents. There are also a few private rooms set apart, into which patients are admitted for a weekly payment of 1½ to 2 guineas. This is a great boon to foreigners who are away from their families and cannot have proper nursing in their apartments, as well as to those who come on a visit to the metropolis and have the misfortune to fall ill, and would, but for this accommodation, have to remain in hotels at great inconvenience and expense. The grand total of the expenditure amounts to about £9,200, whilst the more reliable income of the hospital, derived from the real and funded property, annual subscriptions, Hospital Sunday and Saturday Funds, &c., amounts to about £5,600, leaving £3,600 to be collected by the hospital. Secretary, Mr. Chr. Feldmann; Matron, Miss Christiane Bürger.

**Great Northern Central Hospital**, Holloway Road, N.—This poor, free, and unendowed charity is now urgently in want of funds both to meet the ordinary expenses of maintenance and also to enable the Committee to complete the buildings. Although the ordinary expenses have considerably increased during the last two or three years, owing to the greater amount of work done, the receipts have diminished, and at the present time the necessary funds to pay the bills for last quarter which will soon be due, are still wanting. The yearly income derived from annual subscriptions and interest is about £1,800, while the expenditure is £6,000; the institution, therefore, is mainly dependent on such uncertain sources of income as donations and legacies. £27,000 at least is required to finish the buildings, the first part of which was opened in 1888. Since that date appeals have been made continuously for contributions to this sum, but only £8,000 has been received or promised. Yet the additional accommodation is so greatly needed that serious cases have daily to be refused admission for want of room, and others have to wait a long time before they can be received. The second portion of the building is now in course of erection, and money is urgently required to meet the calls of the contractors. H.R.H. the Duke of York will preside at a festival dinner in February next, and gentlemen willing to act as stewards are invited to send their names to the Secretary. The Committee appeal most earnestly for the means to carry on their all-important work. Secretary, Mr. William T. Grant; Matron, Miss M. Hull.

**Guy's Hospital**, Borough, S.E.—Guy's is too old a friend of the public to need much said in its favour, only so long as it has closed wards and is crippled by poverty it is a reproach to the metropolis. The net income of the hospital derived from its landed estates remains at £14,000 per annum less than it was before the agricultural depression, with no present prospect of improvement. There is left, therefore, only an assured income of £25,000 to maintain the

500 beds now open, and which cannot be kept up at a less cost than £38,000 per annum. The greater part of this deficiency can only be met in the future as in the past, by liberal contributions from the public, exclusive of any further aid from the same source, required to re-open 100 beds still remaining vacant, for which there is a pressing demand. Matron, Miss V. Jones; Superintendent, Dr. Perry.

**King's College Hospital**, Portugal Street, W.C.—That this hospital has much claim on the public for the good work it does goes without saying. Its finances are not, however, in that flourishing condition which they ought to be in, were the hospital supported as it should be. First, there is a need for money to meet the current expenses, as of assured income it has but £750 to meet an expenditure of £17,000. Next comes the need of subscriptions towards paying off a loan of £3,500, due to the bankers; and thirdly, were the money needed to pay off this loan forthcoming, the really much-needed improvements to the out-patients' department could be carried out. Recently some old houses close up against the hospital, excluding light and air, have been pulled down and the new Bankruptcy Offices built on the site at a fair distance from the hospital windows. We must draw special attention to the convalescent home, which secures a longer period of ease for the patients sent to it from the hospital wards. For an expenditure, therefore, of about £1,000, the improvements could be carried out. As considerably over 21,000 patients are yearly admitted to the benefits of the institution, it should surely have brought its claims for aid to the doors of many of the giving public, who, now knowing its needs, will, we feel confident, do their utmost to assist it Secretary, Rev. N. Bromley; Sister-Matron, Miss Monk.

**London Homœopathic Hospital**, Great Ormond Street, W.C.—This hospital has a well-earned reputation for efficiency and careful management. During the present year the number of in-patients has been greater than in any previous year; and the out-patients have been more numerous also. The demand for the nurses trained in the hospital continues to increase. Connected with it is the Homœopathic Convalescent Home, 66, Enys Road, Eastbourne. This was established to give to men, women, and children who have recovered from illness the benefits of a temporary Eastbourne home, with good food and careful attention. Patients contribute 7s. per week. Secretary, Mr. G. A. Cross; Lady Superintendent, Miss Brew.

**London Hospital**, Whitechapel Road, E.—This is the greatest hospital in this country. From this fact, and from the fact that it has been persistently attacked by a small clique, in spite of their contentions having been shown to be ill-founded over and over again, we make no doubt that the public will give largely to its funds, in testimony of their appreciation of the enormous value of the work which it does for the poor of East London. It includes special departments under eminent medical men for the treatment of all classes of disease, the number of beds devoted to children being greater than those to be met with in most children's hospitals. The character of the work done and its value to the public may be realised from the statement that the able Matron, Miss Lükes, has three assistants under her, in addition to two night superintendents, 19 day sisters, and 220 staff and probationer nurses. This great hospital of the East-end is in serious want of funds, as the committee depend on voluntary contributions for £50,000 a-year to enable them to maintain the 640 beds which are daily occupied by urgent cases. House Governor and Secretary, Mr. G. Q. Roberts; Matron, Miss Eva C. Lükes.

**Metropolitan Hospital**, Kingsland Road, N.E.—The Metropolitan Hospital, founded in 1836 in the City, and removed in 1887 to the Kingsland Road, as affording wider scope for work, has, within the radius of a mile, a dense and very poor population of over a quarter of a million, untouched by other general hospitals, with the exception of the German Hospital in Dalston Lane. As an instance, it is enough to say that the London on the south-east, St. Bartholomew's on the south-west, and the Great Northern Central on the north-west, are all just two miles away. In four years, in the new building, 78 beds have been opened (out of a possible 160), in which last year 709 in-patients were treated, and the out-patient attendances numbered more than 66,000. It is to be hoped that the Christmas appeal this year will be successful, or beds must be closed in order to curtail the expenditure, £12,000, in round numbers, being required to carry on the work, even on its present insufficient footing. Secretary, Mr. C. H. Byers.

**Miller Hospital**, Greenwich Road, S.E.—Opened in 1885 as a hospital in memory of the late Canon Miller, D.D.; this was the first hospital built in England with circular wards. During the year over 12,000 cases were attended at the hospital, which is entirely dependent on voluntary contributions. About £800 more is required to carry on the work at present. Secretary, Mr. James Marks; Matron, Miss Clara Purvis.

**Middlesex Hospital**, Mortimer Street, W.—This institution undoubtedly does a large share in the beneficent work of relief carried on so courageously by our voluntary hospitals despite all the difficulties in their way. This is at once apparent when we say that in its 307 beds last year 3,109 patients were treated, whilst upwards of 38,000 out-patients received, free of charge, over £1,300 worth of medicines and dressings alone. The cancer wards are a distinguishing feature of the hospital, 34 beds being specially set aside for these cases, and the long care and general treatment required for sufferers from this terrible disease add greatly to the expenses of the hospital. Secretary, Mr. F. Clare Melhado; Lady Superintendent, Miss Thorold.

**North-West London Hospital**, Kentish Town Road, N.W.—This is the only institution of the kind in the north-west district, and it is situated in the midst of a poor and densely-populated neighbourhood. Though wards for adults have been opened since its establishment, in order to try and meet the great need for a general hospital in the district, the special object for which the institution was founded, viz., the treatment of sick children, continues to be its chief characteristic. Not only has the hospital been an inestimable boon to the immediate neighbourhood, but many sufferers from different parts of the kingdom have shared in its benefits. One special feature in this charity is the treasurer's soup kitchen, which is open during winter, and provides upwards of 2,000 soup dinners per week. Additional annual subscriptions are specially needed to provide a more permanent income. Secretary, Mr. Alfred Craske.

**Poplar H·spital for Accidents,** Blackwall, E.—This hospital is placed in a position in the far East-end of London, close t͡o the Dock gates, close to great iron works, close to the largest gas works in London, and to many other large works. More accidents occur in this district than in any other, and the hospital is an absolute necessity. Yet during this last year many men and children requiring immediate admission and attention have had to be refused owing to want of room. This refusal means the carrying of a man, often with broken limbs or fractured skull, for over two miles to the nearest hospital, *i. e.* the London, however severely injured. Added to this, there is no women's ward, and very little accommodation for children. The rebuilding of the hospital has become a necessity, and the Committee have, therefore, determined to leave no efforts unturned and to appeal to the public to help generously; £4,(00 has been already received towards rebuilding and additions, but it will cost £5,000 more to complete all that is needed, and to found the nucleus of an endowment fund. Secretary, Lieut.-Colonel Feneran; Matron, Miss E. Pilcher.

**Royal Free Hospital,** Gray's Inn Road, W.C.—For many years no special appeal has been made by this hospital to the public for funds; now, however, the Committee have issued an appeal for contributions to enable them to rebuild the front portion of the hospital, which is the only part of the old buildings (formerly used as barracks) now remaining, and which is in striking and painful contrast to the completeness of the more modern portions. This is not done for the sake of ornamentation, but because the departments situated in this portion of the hospital are antiquated and quite inadequate, and the structure is so dilapidated that it is useless to further attempt to repair it. With regard to the claims of the hospital on the generous support of the public, it may be observed that since its foundation it has admitted the sick and suffering poor to the free and unrestricted enjoyment of its benefits without letters of recommendation being required from subscribers or others, poverty and sickness being the only passport required. This principle has since been more or less adopted by other hospitals in London, but the Royal Free Hospital is still the only one in which it is acted upon in its entirety. Secretary, Mr. Conrad W. Thies.

**St. George's Hospital,** Hyde Park Corner, S.W.—Occupying a commanding position, there is reason to believe that it suffers in consequence, and we hope that all who pass St. George's Hospital in future will contribute something to its funds in the course of the year. Secretary and Superintendent, Mr. C. L. Todd; Matron, Mrs. Coster.

**St. Mary's Hospital,** Paddington, W.—About 2,000 men, 1,500 women, and 500 children are treated as in-patients at St. Mary's during the year, while about 20,000 persons attend the out-patient department yearly. To meet the expenditure caused by this enormous relief of suffering (some £22,000), the hospital has an assured income of about £2,500, so that nearly £20,000 every year has to be collected from the charitable. Besides having to find this large sum of money, the Committee are endeavouring to raise £50,000 to defray the expense of the new wing on the Praed Street side of the hospital, the foundation stone of which has just been laid by the Prince of Wales, and which will materially add to the hospital. Secretary, Mr. Thomas Ryan; Matron, Miss Medill.

**Seamen's Hospital (Drea[illegible]nought) Society,** Greenwich, S.E.—The special needs of this society at the present time are to find support for the new branch hospital at the Royal Victoria and Albert Docks, and to support the increasing number of patients in the wards at Greenwich.

This branch, which acts as a feeder for the hospital at Greenwich, and has had the effect of greatly increasing the average number of patients in the wards, entails an expenditure of over £1,200 per annum, and to this must be added the extra cost of the increased number of patients at Greenwich. Roughly, it may be stated that the requirements of the society, with its two hospitals and two dispensaries, are £2,000 in excess of what they were two years ago, while, on the other hand, very little additional support has been accorded to the society. Secretary, Mr. P. Michelli; Matron, Miss Cooke.

**University College Hospital,** Gower Street, W.—The North London or University College Hospital is mainly dependent upon voluntary contributions, the income on which it can rely being only £6,000, whilst the necessary annual expenditure is very nearly £20,000. The rebuilding of the hospital, too, upon a more modern plan, as soon as possible, is an urgent necessity, and for this purpose a fund was instituted in 1884, named the "Jubilee Endowment and Building" Fund, to which contributions are earnestly invited. Secretary, Mr. Newton H. Nixon.

**Westminster Hospital,** Broad Sanctuary, S.W.—Only £2,710, out of an expenditure of £15,000, is assured to this charity, so that about £12,000 has to be made up each year in subscriptions. It is estimated that there will be a deficiency this year of about £3,500. Not only is the hospital of use to those resident in the immediate neighbourhood, but as some 8,000 casualties are received within its walls, it may be inferred that others ought to take an interest in its wellbeing. It does an immense service to the country by training a large number of excellent nurses. Secretary, Mr. Sidney M. Quennell; Matron, Miss Pyne.

**West London Hospital,** Hammersmith Road, W.—This is the nearest hospital for a population of nearly 500,000 persons. The 101 beds are utterly disproportionate to the needs of this district, so that it is now quite an ordinary event for eight suitable cases in a single day to be unable to gain admission. The committee have purchased, at a cost of nearly £7,000, some adjoining freehold property, and now appeal for £40,000 wherewith to provide thereon adequate accommodation. Secretary, Mr. R. J. Gilbert; Lady Superintendent, Miss Hardy.

## Figures and Tables

**Figure f1:**
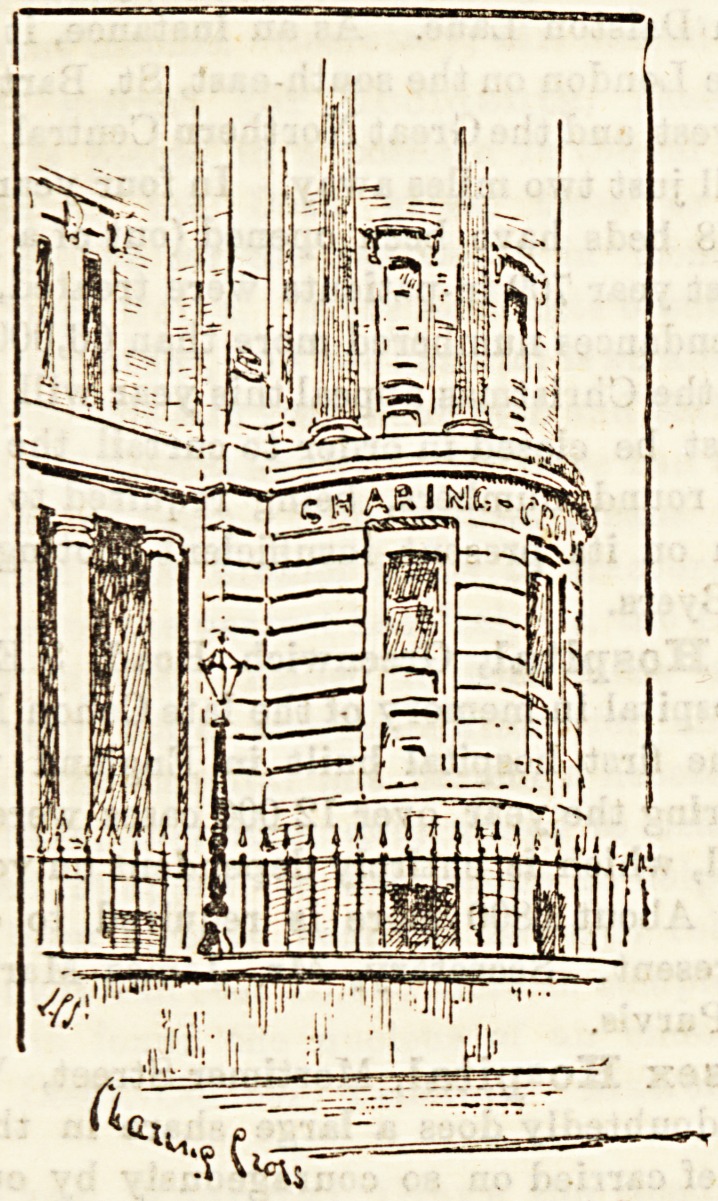


**Figure f2:**
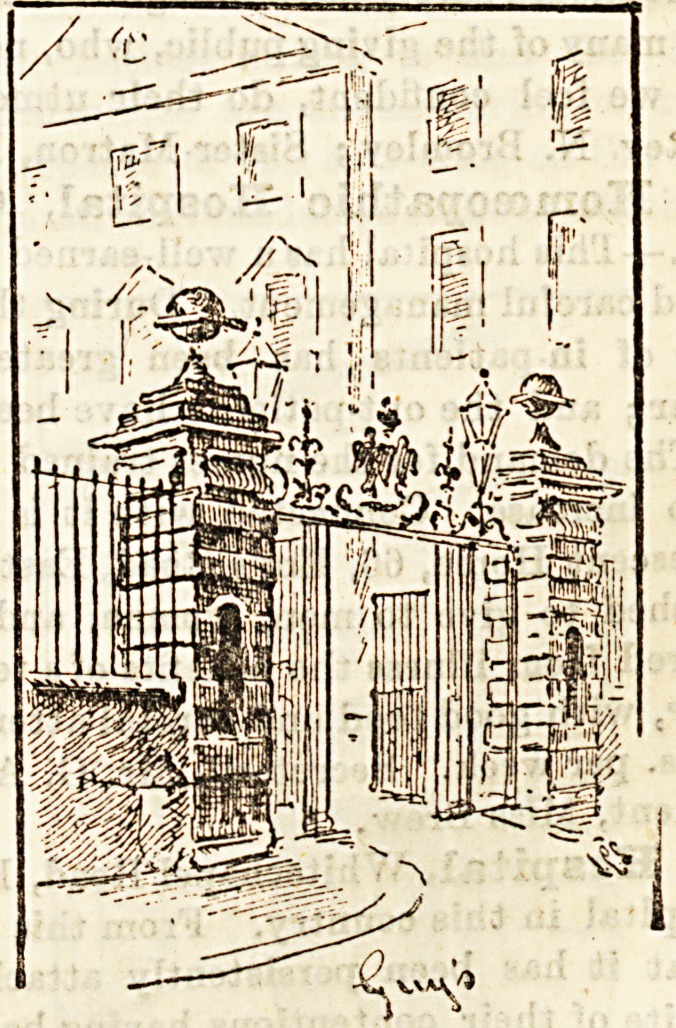


**Figure f3:**
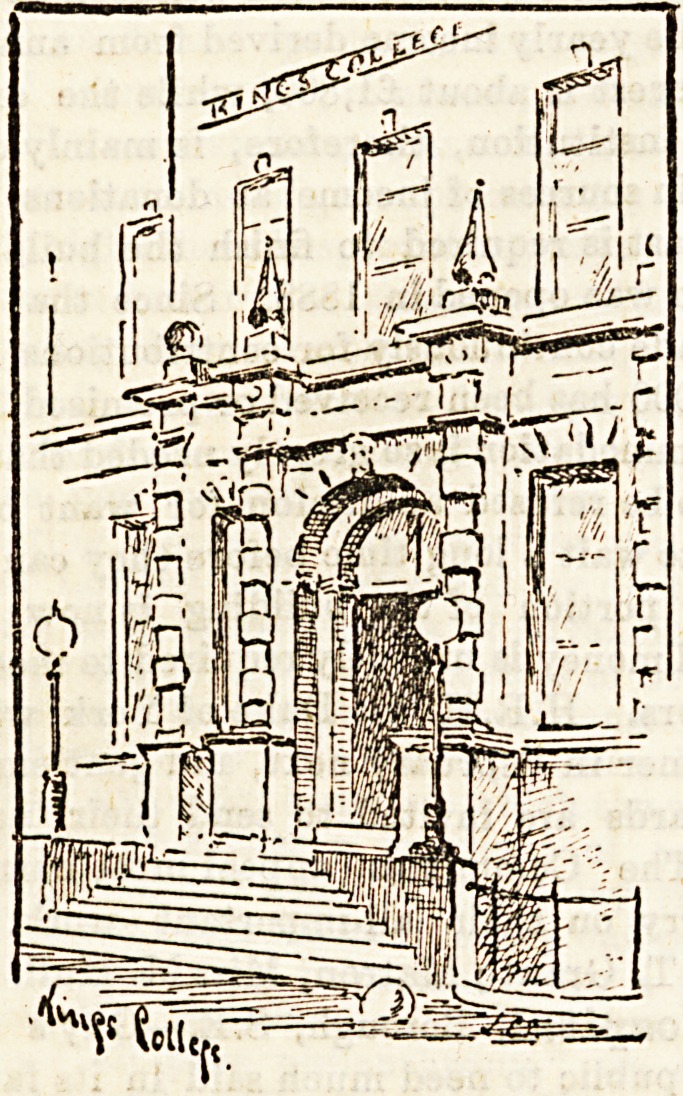


**Figure f4:**
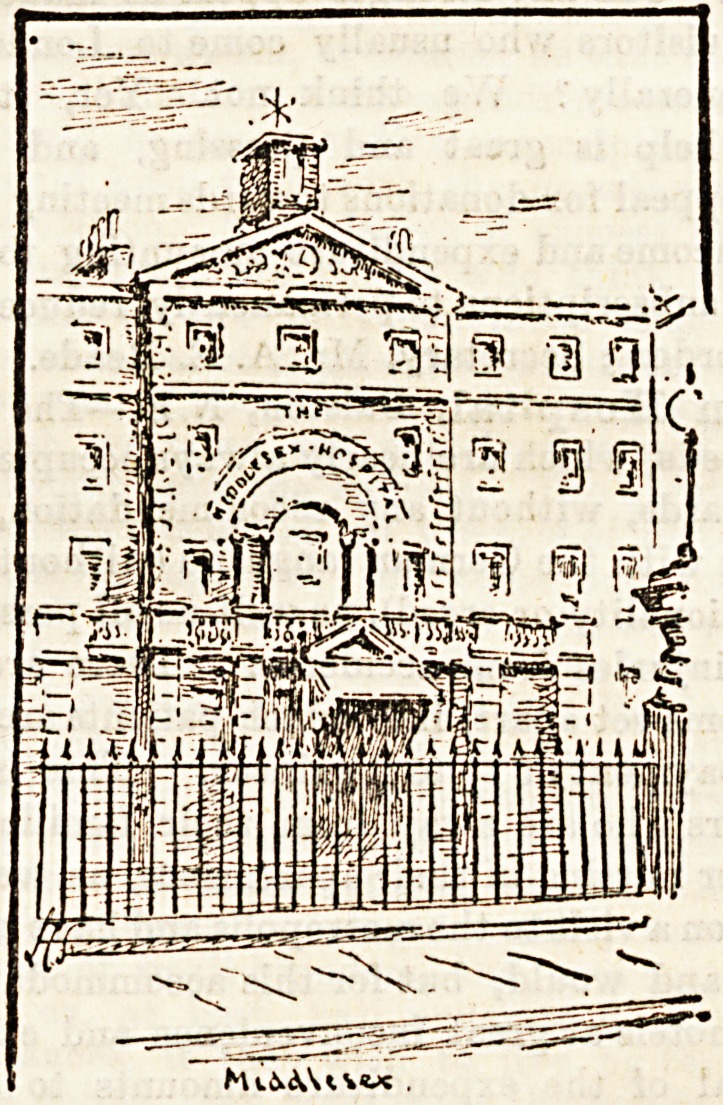


**Figure f5:**
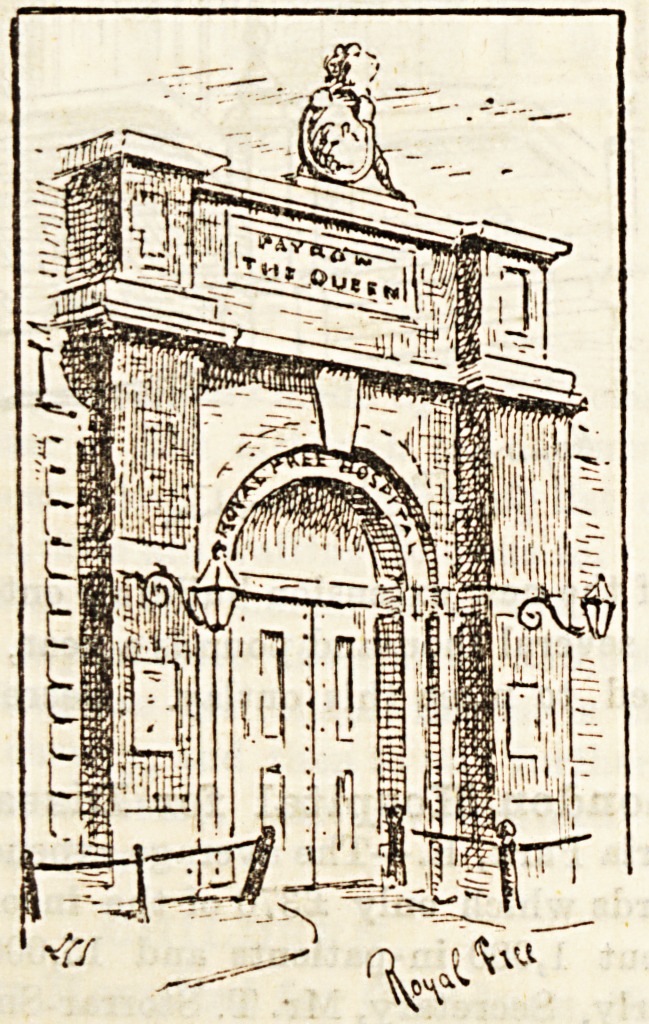


**Figure f6:**
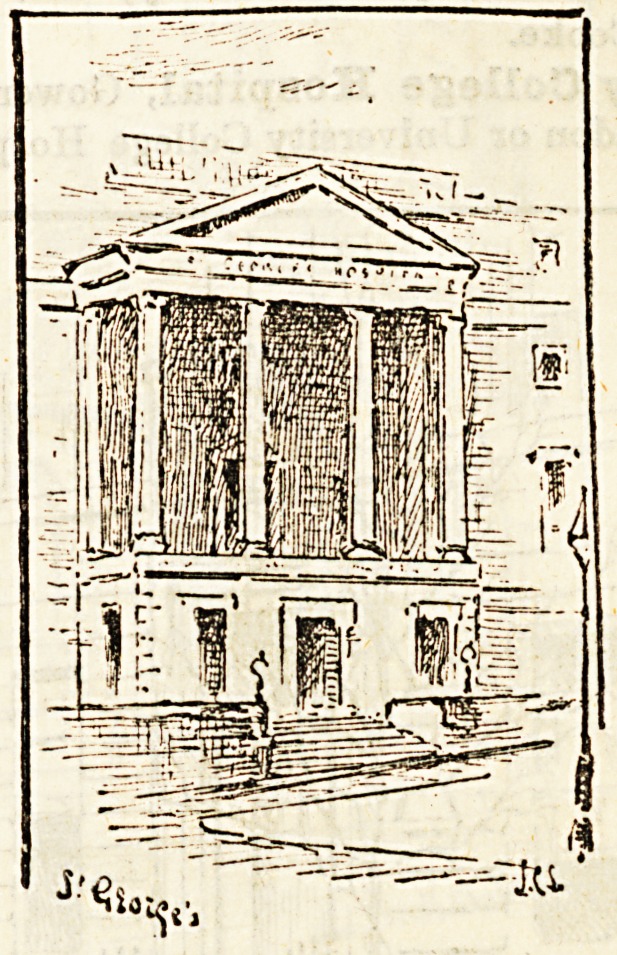


**Figure f7:**
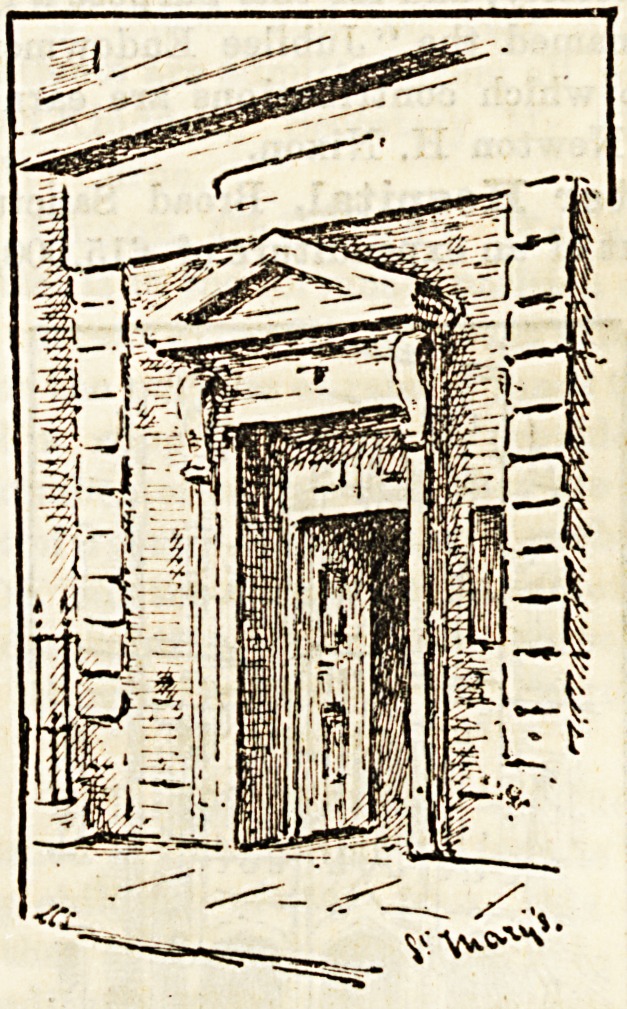


**Figure f8:**
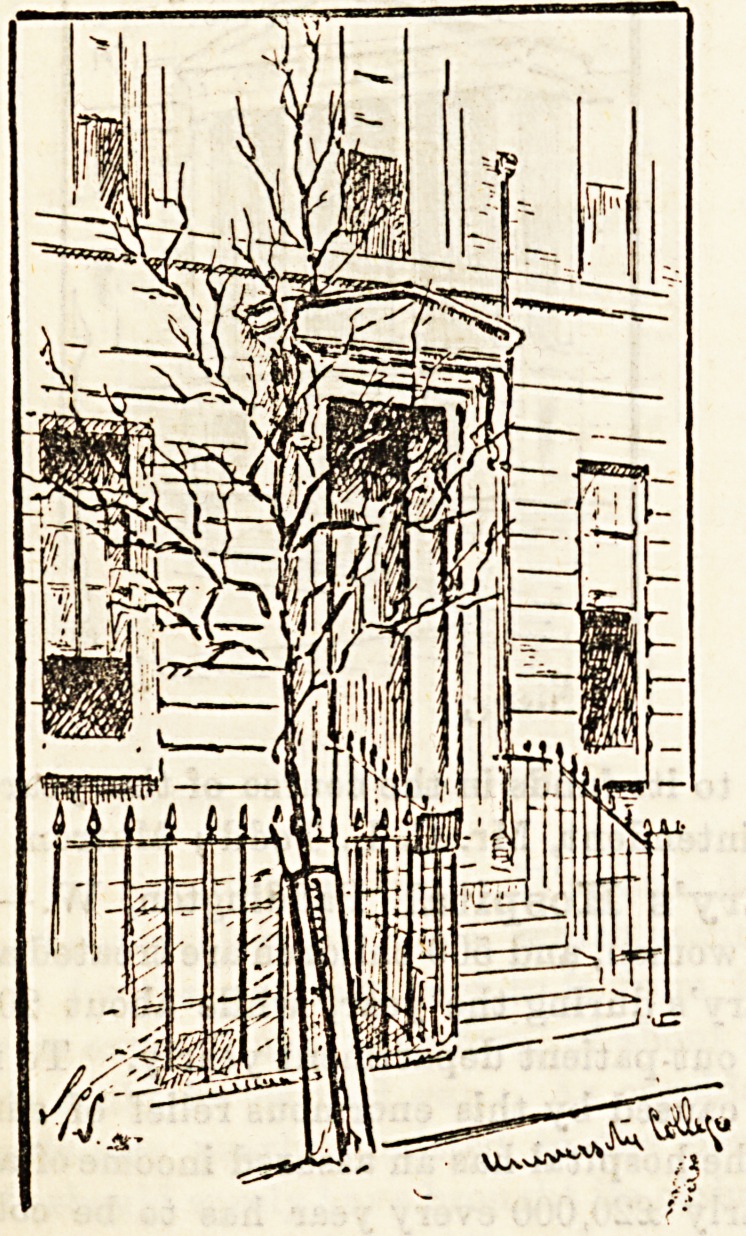


**Figure f9:**